# Glucocorticoids induce osteonecrosis of the femoral head in rats via PI3K/AKT/FOXO1 signaling pathway

**DOI:** 10.7717/peerj.13319

**Published:** 2022-05-03

**Authors:** Fei Sun, Jian Lin Zhou, Si Xing Wei, Ze Wen Jiang, Hao Peng

**Affiliations:** Renmin Hospital of Wuhan University, Wuhan, China

**Keywords:** SONFH, Glucocorticoids, Dexamethasone, Osteoblast, Apoptosis, PI3K/AKT, FOXO1

## Abstract

**Background:**

Steroid-induced osteonecrosis of the femoral head (SONFH) is a disorder that causes severe disability in patients and has a high incidence worldwide. Although glucocorticoid (GC)-induced apoptosis of osteoblasts is an important cytological basis of SONFH, the detailed mechanism underlying SONFH pathogenesis remains elusive. PI3K/AKT signaling pathway was reported to involve in cell survival and apoptosis.

**Objective:**

We explored the role of PI3K/AKT/FOXO1 signaling pathway and its downstream targets during glucocorticoid -induced osteonecrosis of the femoral head.

**Methods:**

We obtained gene expression profile of osteoblasts subjected to dexamethasone (Dex) treatment from the Gene Expression Omnibus (GEO) database. Differentially expressed genes (DEGs) were screened out and functional enrichment analysis were conducted by bioinformatics analysis. *In vitro*, we analyzed Dex-induced apoptosis in MC3T3-E1 cells and explored the role of PI3K/AKT/FOXO1 signaling pathway in this phenomenon by employing siRNA-FOXO1 and IGF-1(PI3K/AKT agonist). Finally, we verified our results in a rat model of SONFH.

**Results:**

In Dex-treated osteoblasts, DEGs were mainly enriched in the FOXO signaling pathway. Dex inhibited MC3T3-E1 cell viability in a dose-dependent effect and induced apoptosis by increasing the expression levels of FOXO1, Bax, cleaved-Caspase-3, and cleaved-Caspase-9, while reducing the expression of Bcl-2. Notably, these results were reversed by siRNA-FOXO1 treatment. Dex inhibited PI3K/AKT signaling pathway, upregulated FOXO1 expression and increased FOXO1 nuclear translocation, which were reversed by IGF-1. Compared to normal rats, the femoral head of SONFH showed increased expression of FOXO1, increased number of apoptotic cells, and empty osteocytic lacunas, as well as decreased bone tissue content and femoral head integrity. Significantly, the effects of GC-induced SONFH were alleviated following IGF-1 treatment.

**Conclusion:**

Dex induces osteoblast apoptosis via the PI3K/AKT/FOXO1 signaling pathway. Our research offers new insights into the underlying molecular mechanisms of glucocorticoid-induced osteonecrosis in SONFH and proposes FOXO1 as a therapeutic target for this disease.

## Introduction

Administration of glucocorticoids (GC) is the most common risk of non-traumatic femoral head necrosis (Chang, Greenspan & Gershwin, 2020; Yoon et al., 2019). GC exert strong anti-inflammatory and immunosuppressive effects and are widely used in clinical settings. However, GC can cause various complications in the skeletal system, including osteoporosis and osteonecrosis (Caplan et al., 2017; Al-Omari et al., 2020; O’Brien et al., 2004). SONFH causes severe disability in patients and has a high incidence. Osteocytes apoptosis induced by a high-dose/short-term or low-dose/long-term GC administration is an important cytological feature for SONFH (Larson et al., 2018; Weinstein, 2012a; Thompson & Ensrud, 2020). Hence, studying the underlying mechanisms of GC-induced bone cells apoptosis will unravel pathogenesis and progression for SONFH.

Forkhead box-O1 (FOXO1), an important downstream target of AKT (Wang et al., 2020), is a transcription factor involved in regulating several intracellular life targets, including expression of genes encoding molecules involved in apoptosis, energy metabolism, immune modulation, and anti-oxidative stress (Xing et al., 2018). In addition, several genes acting upstream of FOXO1 regulate its expression and function in numerous biological processes, which makes it a pivotal transcription factor (Peng et al., 2020). Hence, the regulation of FOXO1 might provide a crucial clue for devising disease preventing and therapeutic strategies.

With the advent of high-throughput gene sequencing technology, bioinformatics is increasingly being used for elucidating gene expression profiles and exploring potential genetic mechanisms underlying various disorders (Jin et al., 2021; Gauthier et al., 2019). In medical research, gene expression profiles have been widely used to identify DEGs, which can help to explore the mechanisms of gene action in various diseases.

In this study, we used bioinformatic tools to predict important signaling pathway in Dex-treated osteoblasts. We conducted *in vitro* experiments to explore the function of PI3K/AKT/ FOXO1 signaling pathways and its downstream targets on Dex-induced osteoblast apoptosis. Finally, we verified our results using a SONFH rat model. The outcomes of this study enhance the understanding for the molecular mechanism of GC-induced osteoblast apoptosis.

## Materials and methods

### Microarray data collection

We downloaded the raw data (GSE21727 and GSE10311) from the GEO (https://www.ncbi.nlm.nih.gov/geo/) database. These two data sets consist of three untreated human primary osteoblast cells and 3 Dex-treated osteoblast cells, respectively.

### Identification of DEGs

The R package linear models for microarray data (limma) was employed to screen DEGs that met the following cutoff criteria: —logFC— ≥1 and adjusted *P* value <0.05 (Lagerwaard et al., 2021; Liang et al., 2020). All DEGs were visualized as a heatmap and a volcano plot using the “pheatmap” and “ggplot2” packages in R (Version 4.0.2), respectively.

### Functional enrichment analysis of DEGs

To understand the function of DGEs in cell activity, Gene Ontology (GO) and Kyoto Encyclopedia of Genes and Genomes (KEGG) functional annotation were performed by R packages “org.hs.eg.db” and “clusterProfiler”. A GO function enrichment analysis including biological processes (BP), cellular components (CC), and molecular functions (MF) was conducted to identify the biological roles of the DEGs, and a KEGG analysis was conducted to explore the important signaling pathways involved in cellular changes. The significant level of threshold was adj. *P* < 0.05.

### Cell culture and cell viability assays

Murine primary osteoblast MC3T3-E1 cell line was purchased from the Cell Bank of Type Culture Collection of Chinese Academy of Sciences and incubated in Hyclone *α*-MEM medium added with 10% fetal bovine serum (Gibco, USA). The cells were cultured in humidified chamber set at 37 °C and supplemented with air containing 5% CO2. Cell Counting Kit-8 (CCK-8, Abcam, ab228554) was used to assess the viability of MC3T3-E1 cells treated with Dex (Sigma-Aldrich, USA). Specifically, MC3T3-E1 cells were seeded in a 96-well plate (2,500 cells per well) and cultured with different concentrations (1, 10, 100, 200, 300 and 400 µM) of Dex for 48 h. Cell viability was determined by a microplate reader at 450 nm of the absorbance value.

### Flow cytometry to detect apoptosis

According to manufacturer’s instructions, Annexiv-FITC Apoptosis Kit (Biotechnology, Beijing, China) was used to detect apoptosis in MC3T3-E1 cells subjected to 48 h Dex treatment.

### siRNA transfection

FOXO1-specific small interference RNA (siRNA) was constructed from Ribobio (Guangdong, China). Cells incubated in a six-well plate were transfected with negative control (siNC-RNA) and siRNA- FOXO1 at optimal concentration (50 nM) in Opti-MEM by the assistant of Lipofectamine 3000 (Invitrogen, USA). The cells were harvested 48 h after transfection and used for subsequent experiments. The detailed sequences of siRNA-FOXO1 are as follows: siRNA1-FOXO1: GCACCGACTTTATGAGCAA; siRNA2-FOXO1: GGACAACAACAGTAAATTT; siRNA3-FOXO1: CAGCAACGATGACTTTGAT.

### Immunofluorescence

MC3T3-E1 cells (10,000 cells/well) were seeded on a 12-well plate and subjected to different treatments (Con, Dex and Dex+IGF-1) for 48 h. In Dex+IGF-1 group, MC3T3-E1 cells were pre-incubated with IGF-1(50 ng/mL; Sigma, USA) for 2 h, and then co-incubated with 200 µM Dex for 48 h. After washed by PBS for three times, cells were fixed with 4% formaldehyde and permeabilized by the assistant of 0.5% Triton X-100. Subsequently, cells were blocked with 5% bovine serum albumin (BSA), co-incubated with the primary antibody (FOXO1) overnight at 4 °C and FITC-conjugated goat secondary antibody the next day. The nuclei were stained with DAPI, and the outcomes were acquired using a fluorescence microscope.

### Nuclear protein extraction

The nuclear protein was extracted according to the manufacturer’s instructions (Solarbio, China). Histone-H3 was used as an internal control.

### Western blotting analysis

The total protein of MC3T3-E1 cells was extracted using the RIPA buffer supplemented protease inhibitors. The protein samples of each group were separated by electrophoresis on SDS-PAGE and transferred to PVDF membrane. Next, the membrane was blocked using 5% BSA at room temperature for 1 h and subsequently incubated with the primary antibody overnight at 4 °C. The following day, the membrane was washed three times using Tris-buffered saline and incubated with corresponding secondary FITC-conjugated goat antibody for 1 h at 4 °C. Finally, the protein bands were recorded using the Bio-Rad ChemiDoc TM XRS system. All the primary antibodies details were as follows: Bcl2(Cat No: 12789-1-AP), Bax(Cat No: 50599-2-Ig), caspase3(Cat No: 66470-2-Ig), caspase9(Cat No: 66169-1-Ig), GAPDH(Cat No: 60004-1-Ig), Histone-H3(Cat No: 17168-1-AP), PI3K (Cat No: 60225-1-Ig), AKT:(Cat No:10176-2-AP), p-AKT (Cat No: 66444-1-Ig), FOXO1(Cat No: 18592-1-AP) were purchased from Proteintech (Wuhan, China). Phospho-FOXO1 (Catalog No. AF4410) and Phospho-PI3K (Catalog No. AF3417) were purchased from Affinity.

### Animal experiments

Eighteen 8-week-old male Sprague Dawley rats (weight 200 ± 20 g) were purchased from Charles River Laboratories (Beijing, China). These rats were housed under conditions of 26 °C, 55% humidity, and a photoperiod of 12 h light and 12 h darkness in an environment free of specific pathogens. All rats were freely access to water and food. These rats were randomly divided into three groups (n = 6/group): (a) Normal group (control), injected with 0.9% saline for 1 week; (b) MPS group, intravenously injected with lipopolysaccharides (LPS, Sigma, USA) 2 mg/kg in the first 2 days, followed by intramuscular injection of methylprednisolone (MPS, Pfizer, USA) 25 mg/kg from day 3–5; (c) MPS+IGF-1 group. IGF-1 was injected intraperitoneally with 1 µg/kg/d 2 h before MPS injection (Chen et al., 2014). On the 30th day, all rats were anesthetized and sacrificed necessarily by intraperitoneal injection of sodium pentobarbital (130 mg/kg) for the collection of the femoral head tissue. The animal experiments were conducted in accordance with the rules of the Laboratory Animal Management and Use Committee of Wuhan University, and approved by the Ethics Committee of Wuhan University (No. 20180920).

### Hematoxylin and eosin (H&E) staining and immunohistochemistry

The rats femoral head were fixed in formaldehyde for 48 h followed by 4-week decalcification in EDTA solution. Next, the decalcified bone tissue was embedded in paraffin and cut into 5 µm-thick sections for H&E staining and immunohistochemical staining (Liu et al., 2017). For immunohistochemical detection, the tissue sections were incubated with the primary antibody of FOXO1 according to the standard protocol. Cells with yellow and brown particles in their cytoplasm and nuclei were identified as positive cells. Three fields of view were randomly selected under the microscope to observe and calculate the proportion of positive cells.

### TUNEL staining

The inSitu Cell Death Detection Kit (Roche, China) was used to evaluate cell apoptosis of rat femoral head tissue with TUNEL staining, according to the manufacturer’s instructions. Three different fields were randomly selected to evaluate the proportion of positive cells.

### MicroCT analysis

We performed micro-CT (Scanco 274 Medical, Switzerland) and thin-slice scan to examine the femoral head with a resolution scale of 12 µm, a scanning voltage of 100 kV, and an electric current of 90 µA. The built-in software of the system was utilized to calculate the following parameters: bone volume fraction (BV/TV), bone trabecular thickness (Tb.Th), bone trabecular space (Tb.Sp), and number of bone trabeculae (Tb.N).

### Statistical analysis

All numerical experiments were performed at least in triplicate. All data are expressed as mean ± SD. Statistical analyses were performed using the GraphPad Prism version 8.0. A paired-samples *t*-test was performed to compare two groups. The multiple comparison test was applied only when the analysis of the variance indicated a significant difference. *P <  0.05, **P < 0.01, *** *P* < 0.0001 was considered statistically significant.

## Results

### Identification of DEGs and functional enrichment analysis

**Figure 1:**
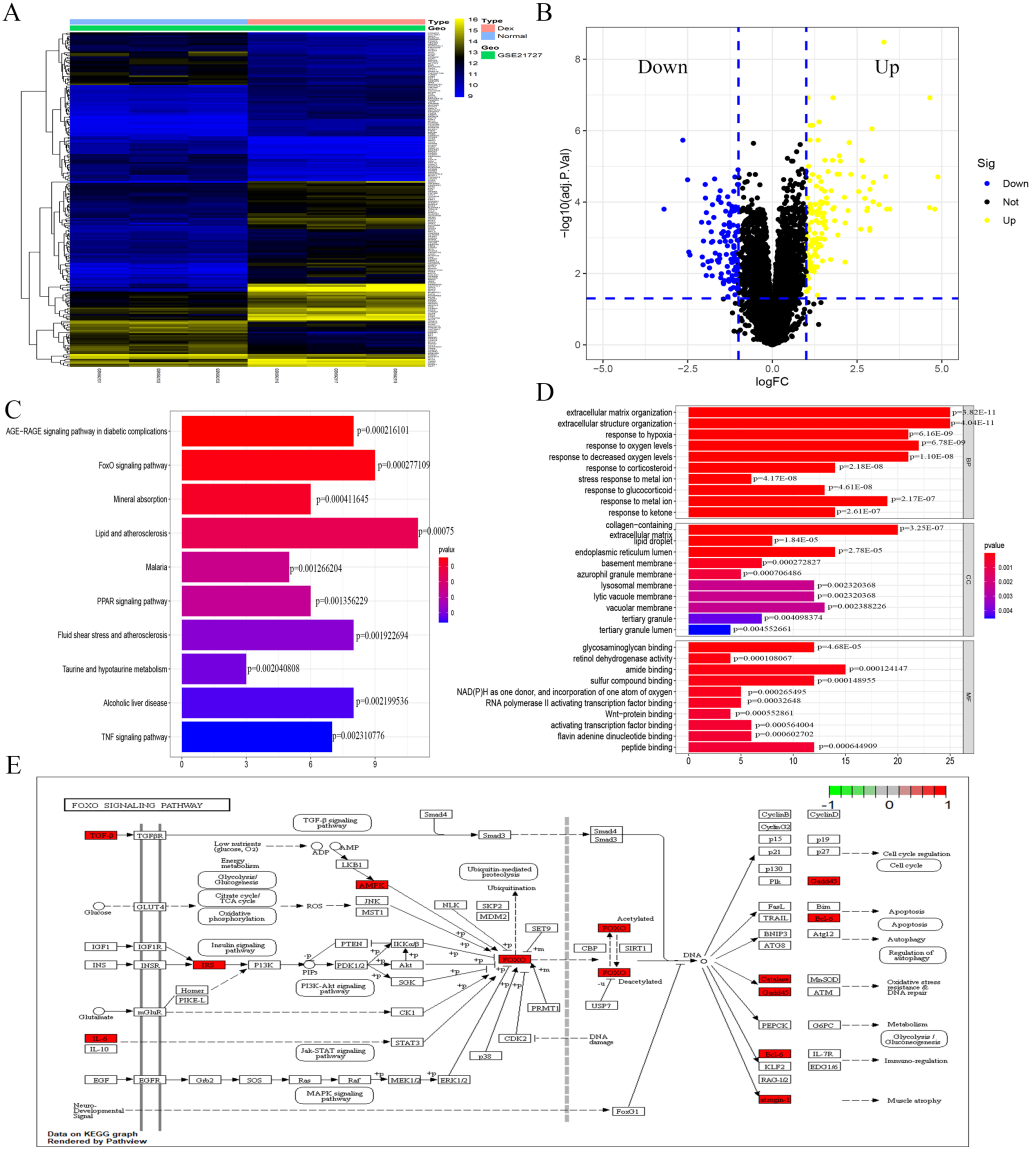
Identification of DEGs caused by Dex from GSE21727. (A) Volcano-plot visualized the DEGs with cut-off criterion as —logFC— >1 and adjusted *P* < 0.05 (red dots means upregulated genes and green dots means downregulated genes). (B) A heatmap of DEGs clustering relationship between control and Dex-treated groups. (C–D) GO and KEGG functional enrichment analysis of DEGs. (E) The function enrichment of the FOXO signaling pathway.

The analysis of the GSE21727 datasets yielded 209 DEGs (DEGs was listed in S1), including 124 and 85 genes with upregulated and downregulated expression, respectively. Figures 1A and 1B show the volcano map and heat map of the DEGs, respectively. During the GO enrichment analysis (Fig. 1C) of the DEGs after Dex treatment, the BP-enriched terms were extracellular matrix organization and extracellular structure organization; the CC-enriched terms were collagen-containing extracellular matrix and lipid droplet; the MF-enriched terms were RNA polymerase II activating transcription factor binding and activating transcription factor binding. The KEGG signaling pathway analysis showed FOXO-mediated pathway as the major signaling pathway (Fig. 1D). The enrichment analysis was performed for DEGs involved in the FOXO signaling pathway (Fig. 1E).

### Dex inhibited MC3T3-E1 cell proliferation and induced apoptosis

**Figure 2:**
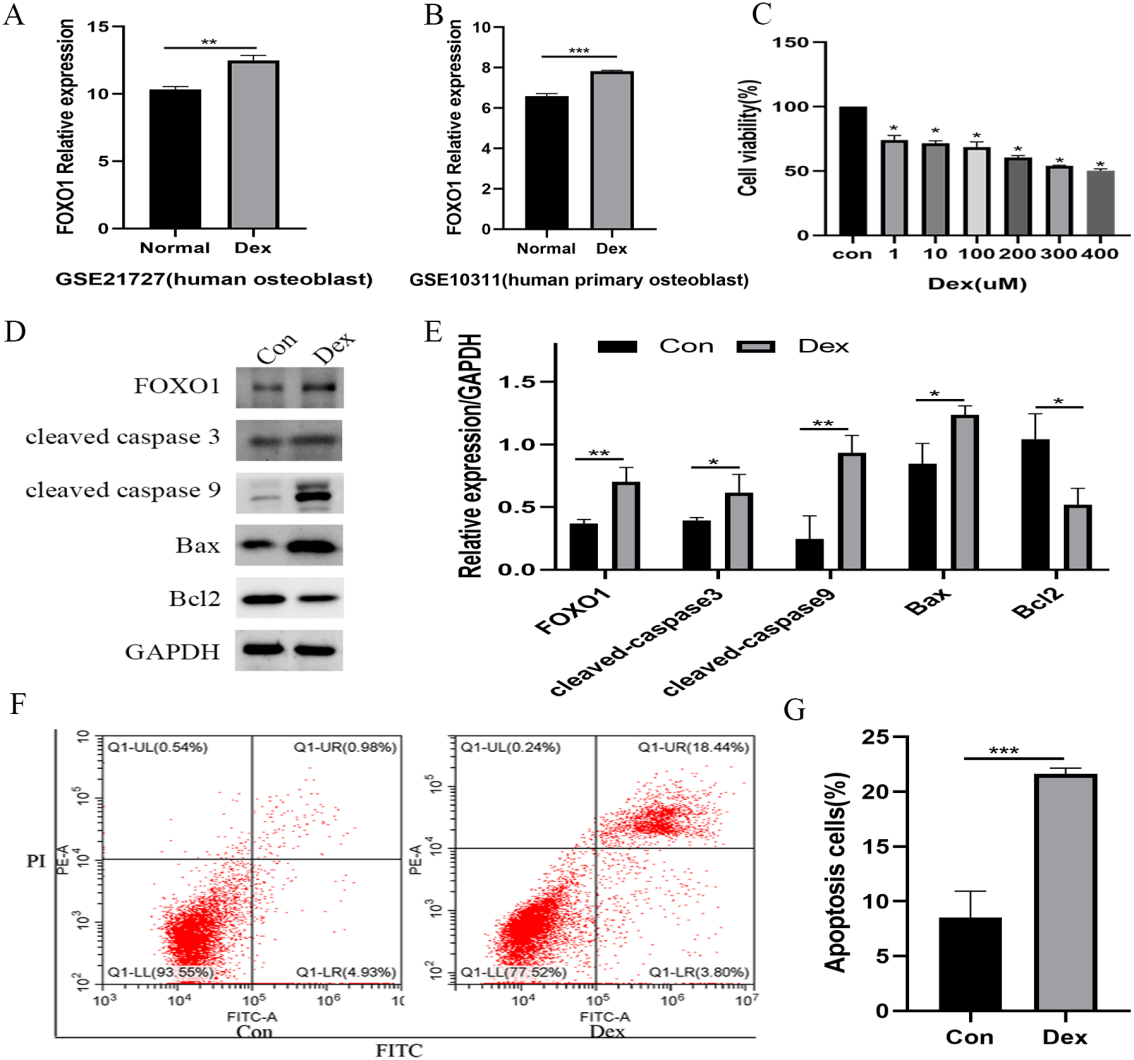
Evaluate the effect of Dex on MC3T3-E1 cells apoptosis. (A–B) The relative expression level of FOXO1 in GSE21727 and GSE10311 from GEO database. (C) Cell viability of MC3T3-E1 after Dex treatment for 48 h. (D–E) Results of western blot for the protein levels of FOXO1, cleaved-Caspase 3, cleaved-Caspase 9, Bax and Bcl-2 after different treatment. (F–G) Flow cytometric analysis of apoptosis rates of MC3T3-E1 cells upon Dex treatment. Con: control group. All data *in vivo* and *in vitro* experiments are triplicate biological replicates. * *P* < 0.05, ** *P* < 0.01, *** *P* < 0.0001.

Following the GSE21727 dataset analysis, FOXO1 expression increased in osteoblasts after Dex treatment (**P = 0.0011, Fig. 2A). Moreover, the FOXO1 expression increased in the validation of GSE10311 dataset (***P < 0.001, Fig. 2B). Results of CCK-8 assay showed that cell viability decreased significantly after 48 h Dex treatment and the IC50 value of Dex was approximately 300 µM (***P < 0.001, Fig. 2C); hence, we chose 48 h treatment with 200 µM Dex for the subsequent experiments. Similar to the microarray dataset analysis, the expression of FOXO1 protein was significantly increased in MC3T3-E1 cells after Dex incubate (**P = 0.0084, Figs. 2D and 2E). Furthermore, compared to the control group, expression levels of cleaved-Caspase-3 (*P = 0.0474), cleaved-Caspase-9 (**P = 0.0068), and BAX (**P = 0.0189) increased after Dex treatment, whereas those of Bcl-2 decreased (*P = 0.0203). The flow cytometry outcomes also showed that the rate of apoptotic cells increased from 8.5 to 21.6% after Dex treatment. These results demonstrate that Dex can significantly increase MC3T3-E1 cell apoptosis (***P = 0.0008, Figs. 2F, 2G, and File S2-Fig. 1).

### Effect of siRNA knockdown FOXO1 expression on Dex-induced apoptosis in osteoblasts

**Figure 3:**
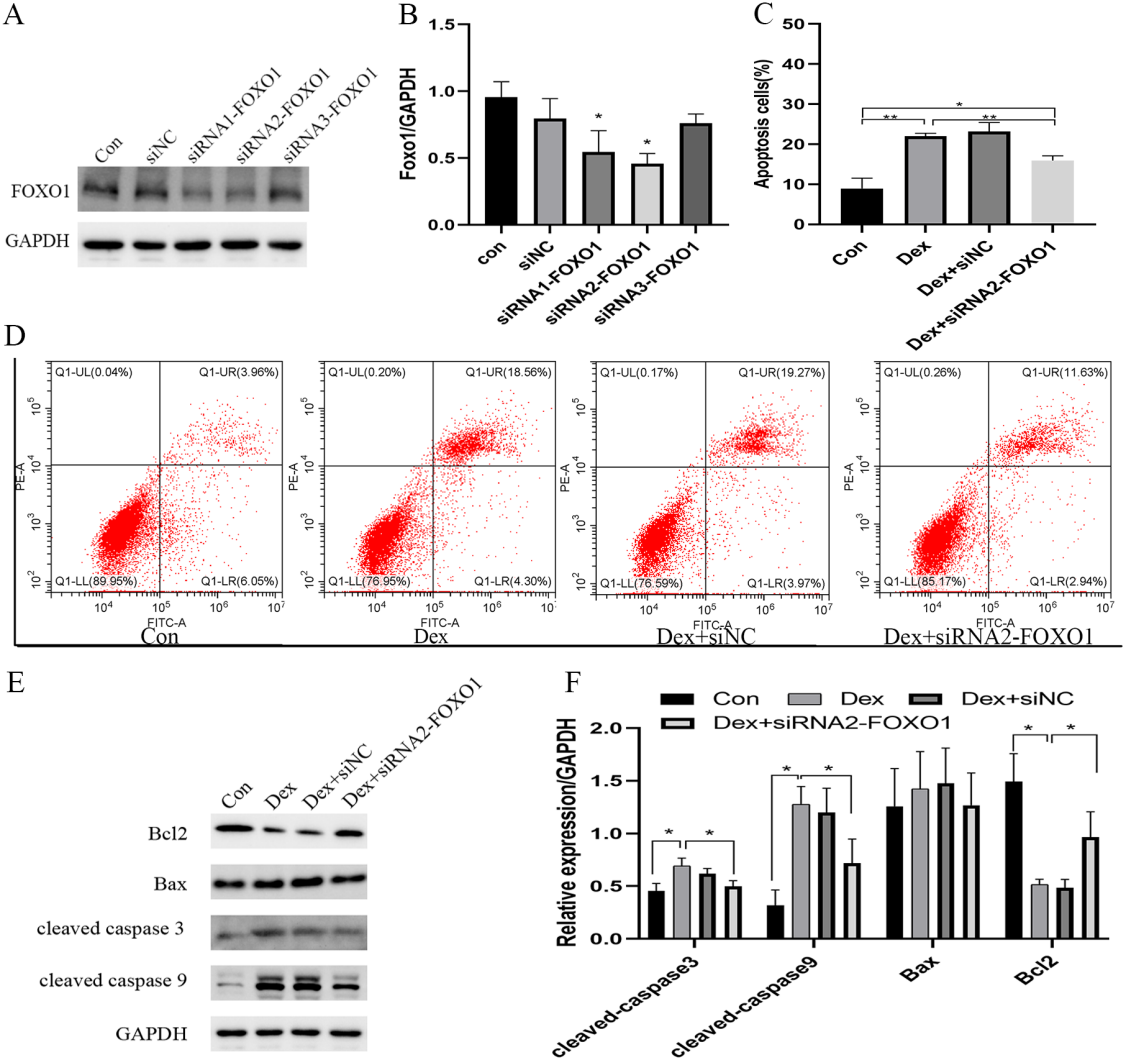
Effect of siRNA-mediated knockdown of Foxo1 on Dex-induced apoptosis. (A–B) MC3T3-E1 cells were transfected with Foxo1 siRNA, and western blotting was performed to analyze the protein levels of FOXO1. (C–D) MC3T3-E1 cell apoptosis after 48 h Dex and siRNA-FOXO1 treatment as determined using FITC-PI/Annexin V staining. (E–F) Protein expression levels of Bax, cleaved-Caspase-3, and Bcl-2 after Dex and siRNA-FOXO1 treatment. GAPDH was used as a loading control. Con: control group. All data *in vivo* and *in vitro* experiments are triplicate biological replicates. * *P* < 0.05, ** *P* < 0.01, *** *P* < 0.0001.

The use of siRNA-FOXO1 (siRNA1-FOXO1 and siRNA2-FOXO1) efficiently reduced the protein expression of FOXO1 (Fig. 3A). Considering the better interference efficiency than siRNA1-FOXO1 (41.4%, ***P = 0.001), siRNA2-FOXO1 (40.6%, **P = 0.0033) was chosen for subsequent experiments (Fig. 3B). The use of siRNA2-FOXO1 inhibited Dex-induced apoptosis (Fig. 3C and File S2-Fig. 2). Specifically, compare with the apoptosis ratio in the control group, the apoptosis ratio of MC3T3-E1 cells was significantly increased (22.86%) in the Dex-treatment group, which was reversed by the effect of siRNA2-FOXO1 (apoptosis ratio as 14.57%, *P = 0.0131, Fig. 3D). Results of western blot revealed that compared to the Dex treatment group, the expression levels of the cleaved-Caspase-3 (*P = 0.0326), cleaved-Caspase-9 (*P = 0.0452), and BAX (*P* = 0.1687) were decreased in the siFOXO1 group, while that of Bcl-2 (*P = 0.0421) increased (Figs. 3E, 3F). These results indicate that siRNA-mediated interference of Foxo1 can reverse Dex-induced apoptosis of MC3T3-E1.

### Dex inhibited the PI3K-AKT signaling pathway

**Figure 4:**
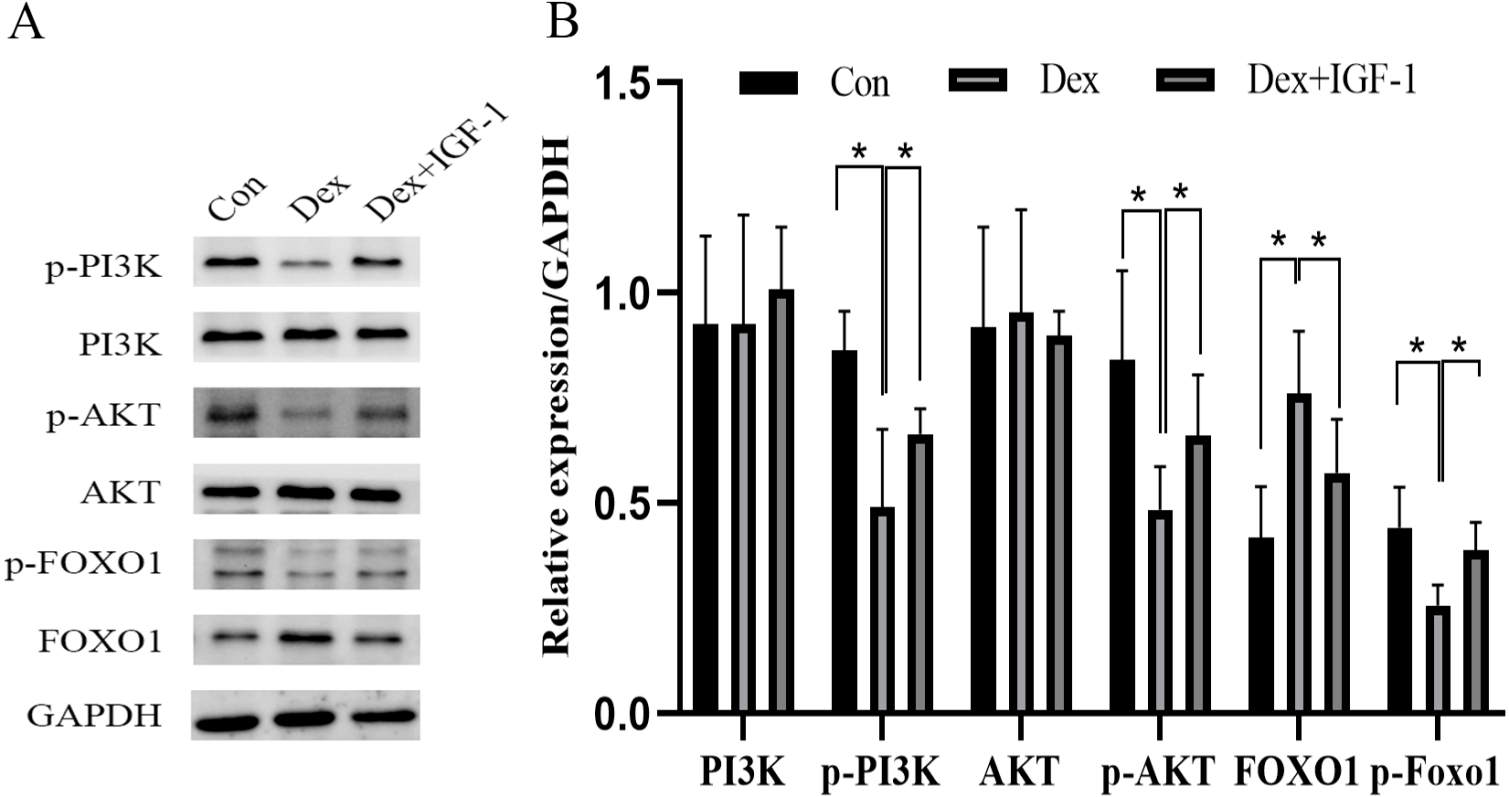
Dex inhibited the activities of PI3K/AKT signaling pathways in MC3T3-E1 cells. (A–B) Western blotting was performed to analyze the expression levels of PI3K, p-PI3K, AKT, p-AKT, FOXO1, and p-FOXO1 in MC3T3-E1 cells after Dex treatment (200 µM, 48 h). Con: control group. All data *in vivo* and *in vitro* experiments are triplicate biological replicates. * *P* < 0.05, ** *P* < 0.01, *** *P* < 0.0001.

Compared to the control group, Dex treatment reduced the expression of p-PI3K (*P = 0.0121) and p-AKT (*P = 0.0376) without affecting their total protein content. Meanwhile, Dex treatment increased the expression of total FOXO1 (*P = 0.0359) while reducing p-FOXO1 (*P = 0.0409) expression. IGF-1 treatment reversed the effects of Dex since compared to the Dex-treated group, the Dex + IGF-1 group showed increased expression of p-PI3K (*P = 0.0437), p-AKT (*P = 0.0308), and p-FOXO1 (*P = 0.0498), while the expression of total FOXO1 (*P = 0.0468) was decreased (Figs. 4A and 4B).

### Dex induced nuclear translocation of FOXO1

**Figure 5:**
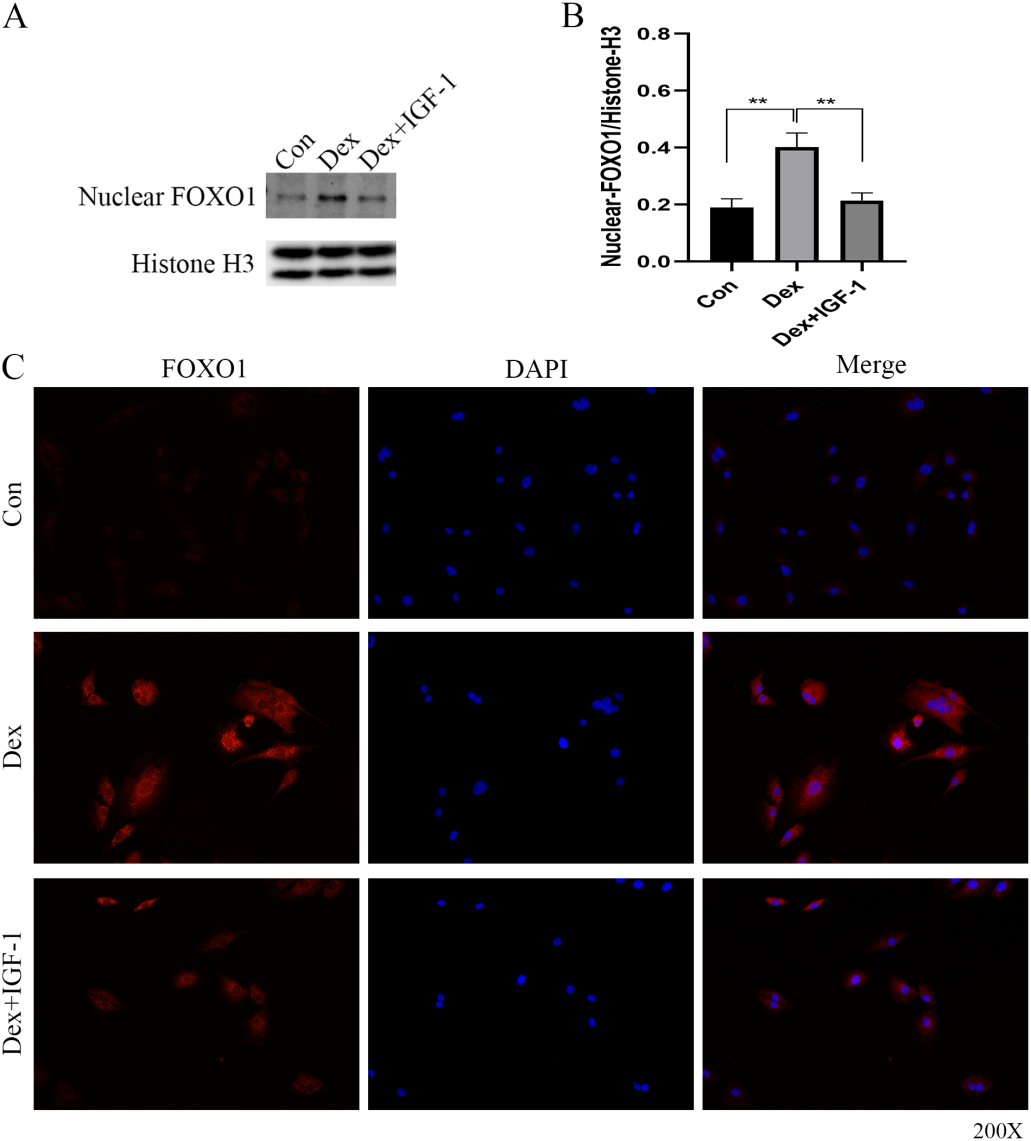
Immunofluorescence studies showing the nuclear translocation of FOXO1. (A–B) Western blotting indicated the expression of FOXO1 in the nucleus after Dex treatment. (C) The distribution and expression of FOXO1 protein in the MC3T3-E1 treated with Dex were evaluated by immunofluorescence. The FOXO1 was stained as red granular dots, while the nucleus was stained with blue. Con: control group. All data *in vivo* and *in vitro* experiments are triplicate biological replicates. * *P* < 0.05, ** *P* < 0.01, *** *P* < 0.0001.

Western blot analysis revealed increased nuclear translocation of FOXO1 after Dex treatment (**P = 0.0032, Figs. 5A and 5B); on the contrary, the nuclear translocation was significantly reduced after IGF-1 treatment (**P = 0.0045). Results of immunofluorescence experiments showed that Dex treatment increased the number of red granular dots in cytoplasm and nucleus of MC3T3-E1 and enhance the intensity of red fluorescence (red dots represent the expression of FOXO1), while IGF-1 may reverse this effect (Fig. 5C and File S2-Fig. 3).

### Animal module results

**Figure 6:**
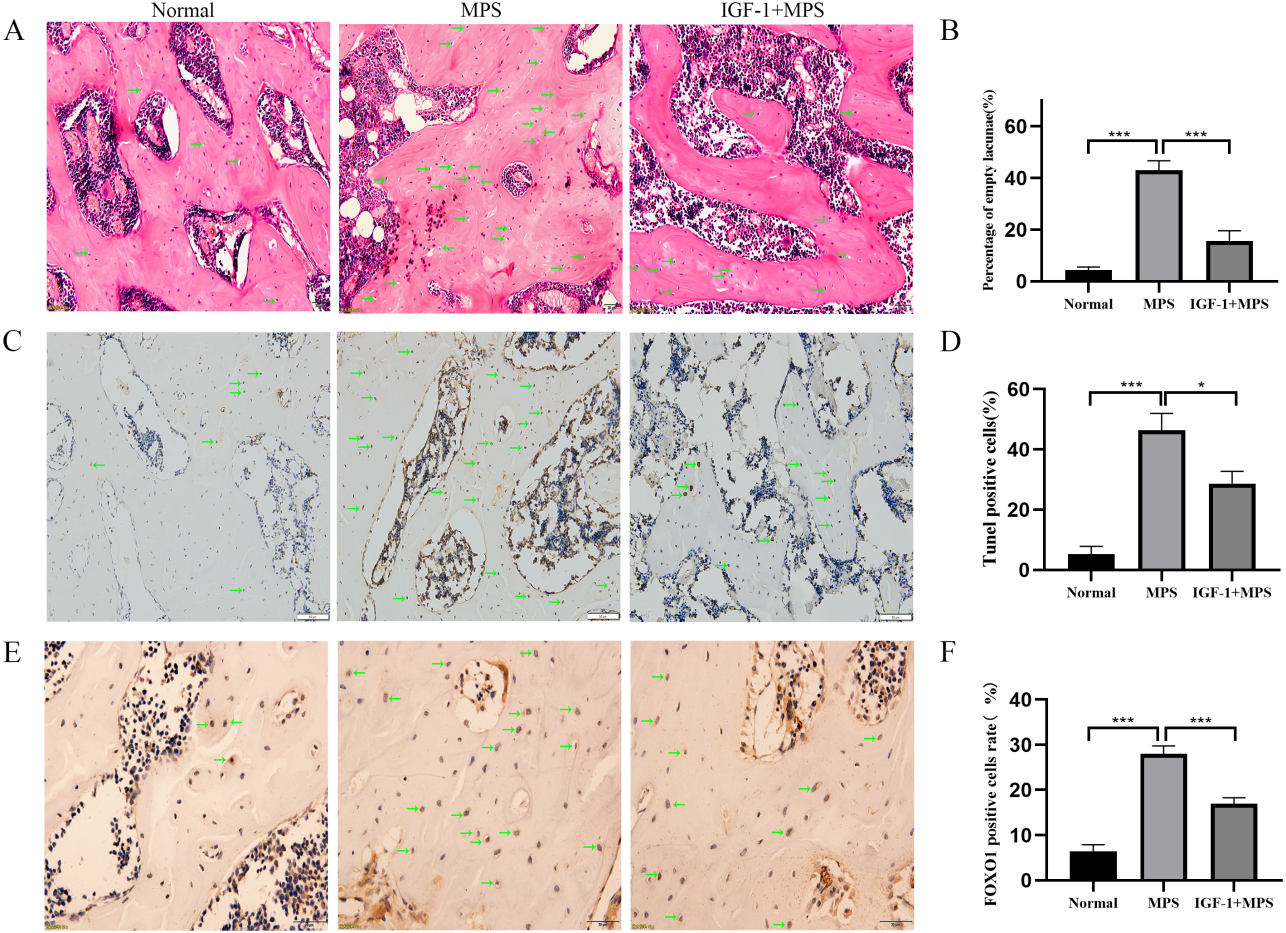
Evaluation of osteonecrosis and apoptosis in animal module. (A–B) HE staining of femoral heads of rats in the Normal, MPS, and IGF-1+ MPS group; green arrows indicate empty osteocytic lacunas. (C–D) Tunel staining to detect apoptosis cells; green arrows indicate positive apoptosis cells. (E–F) Immunohistochemistry to evaluate the expression of FOXO1 in femoral heads of rats; green arrows indicate FOXO1-positive cells that are stained as brown dots. * *P* < 0.05, ** *P* < 0.01, *** *P* < 0.0001.

We conducted *vivo* experiments on rats to validate the effects of GC on inducing the necrosis of femoral head. No necrosis of femoral head was found in the Normal group. Among the six rats in the MPS group, there were four bilateral femoral head necrosis and two unilateral femoral head necrosis (Femoral head necrosis rate as 10/12). Four of six rats in the IGF-1 group were found unilateral femoral head necrosis (Femoral head necrosis rate as 4/12). HE staining of femoral head in normal group rats showed rare empty osteocytic lacunas and intact bone trabeculae (Fig. 6A and File S2-Fig. 4). However, MPS group rats revealed sparse trabeculae and large amount of empty osteolytic lacunas. In IGF-1 group, empty osteocytic lacunas and fractured trabeculae were present but apparently fewer than those in the MPS group. The empty osteocytic lacunas rates in Normal, SONFH, and IGF-1 group were 4.4%, 43.0%, and 15.6%, respectively (Fig. 6B). The apoptosis cells were dyed as brown by TUNEL staining in rat femoral head (Fig. 6C and File S2-Fig. 5). The positive rate of TUNEL staining in Normal, MPS, and IGF-1+MPS group was 5.3%, 46.3%, and 28.6% (Fig. 6D), respectively, which was consistent with the results of HE staining. The level of FOXO1 was increased in MPS group femoral head (***P < 0.001, Figs. 6E and 6F, and File S2-Fig. 6), and decreased by the interference of IGF-1 (***P < 0.001), which was in line with the results of the cell experiment.

**Figure 7:**
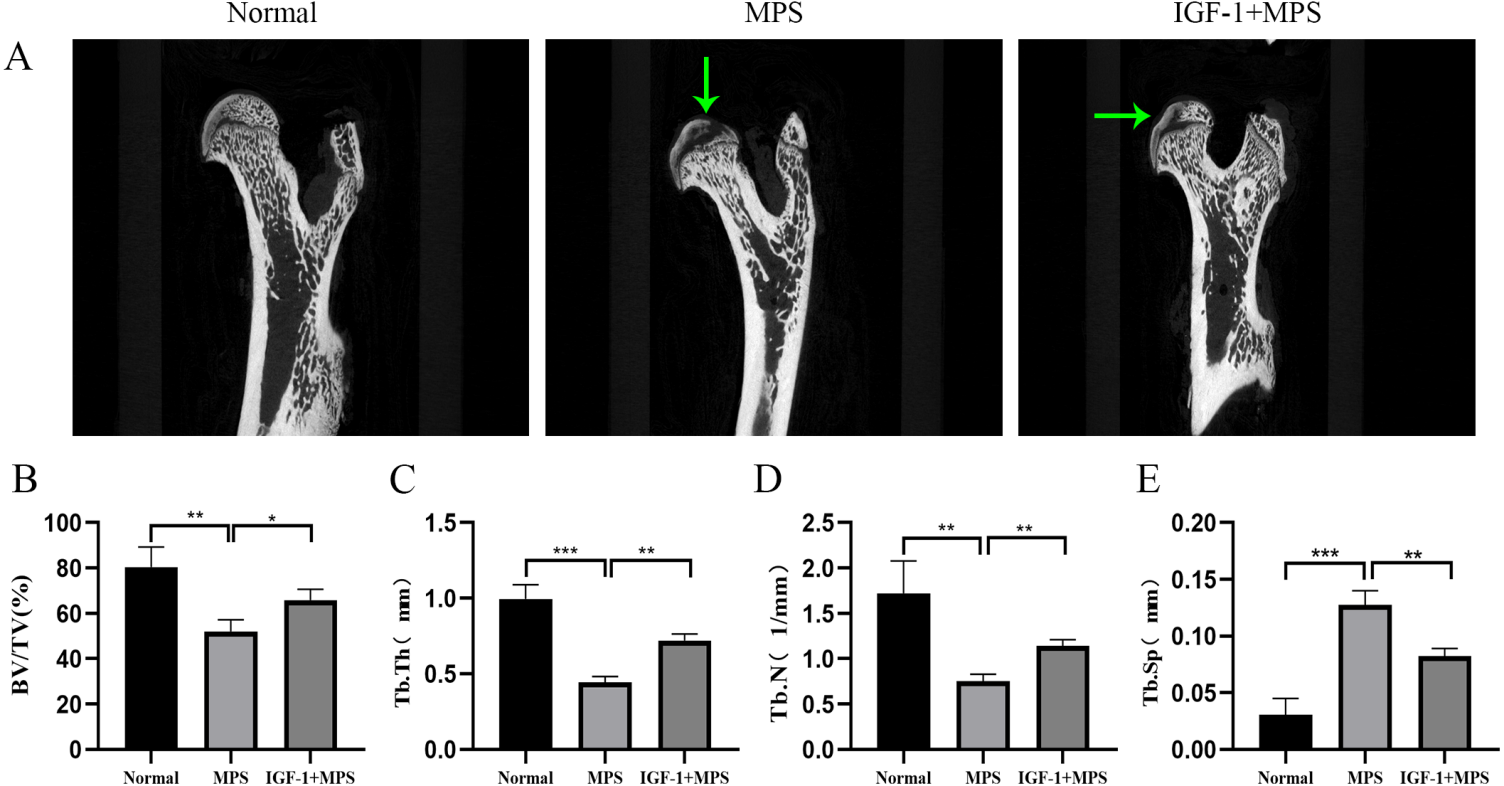
Micro-CT for quantitative evaluation of bone microstructure. (A) Micro-CT scaning for the femoral heads of rats in the Normal, MPS, and IGF-1+ MPS group. green arrows indicate cavity under subchondral bone in the femoral heads of rats. (B–E) Quantitative evaluation of BV/TV(B), Tb.Th(C), Tb.N(D), and Tb.Sp(E) in the three groups. * *P* < 0.05, ** *P* < 0.01, *** *P* < 0.0001.

Micro-CT scanning analysis demonstrated the femoral head of Normal group rats were intact without collapse or cavity, and the bone trabecula was evenly distributed (Fig. 7A and File S2-Fig. 7). Conversely, the MPS group were characterized as obvious cavity under subchondral bone, missing or sparse trabeculae, increased distance between the bone trabeculae (Figs. 7B, 7C, 7D, and 7E). Compared with the MPS group, the femoral head in IGF-1 group have a narrower distance between bone trabeculae (***P = 0.0054), slight collapse and less cavity under subchondral bone.

In short, the results of animal module revealed that compared to the normal rats, the femoral head in SONFH rats showed an increased number of empty osteocytic lacunas, more apoptotic cells, enhanced FOXO1 expression, brittle trabeculae, and decreased bone mass. The use of IGF-1 alleviated the adverse effects of GC on bone destruction in the femoral head of rats.

## Discussion

SONFH disorder causes severe disability in patients, has a high incidence worldwide, and requires hip replacement surgery, making it a burden on the patients and society in general (Seijas et al., 2019; Arbab & Konig, 2016). Osteoblasts, the main functional cell responsible for bone formation, are critical to maintain healthy bone metabolism and prevent osteonecrosis (Han et al., 2018; Sawa et al., 2020). Several studies have demonstrated that osteoblast apoptosis is a key cytological basis for SONFH pathogenesis (Han et al., 2019; Deng et al., 2019; Peng et al., 2021; Weinstein, 2012b). Therefore, it is imperative to understand the pathogenesis caused by GC-induced osteoblast apoptosis to devise better therapeutic interventions. In this study, we analyzed the microarray datasets available on a public database and identified DEGs enriched in FOXO signaling pathway in samples treated with Dex. Moreover, consistent results were obtained using bioinformatics analysis and vitro experiments. Dex can upregulate FOXO1 expression in osteoblasts, thus indicating that FOXO1 plays an important role in intracellular signaling pathways of osteoblasts in response to Dex stimulation.

The activation of FOXO1 may mediate the pro-apoptotic effect of GCs (Zhang et al., 2009; Huang et al., 2019). In this study, Dex treatment upregulated the level of BAX, cleaved-Caspase-3, and cleaved-Caspase-9, but downregulated Bcl-2 level to induce apoptosis of MC3T3-E1 cell. These results were consistent with the outcomes reported by other researches (Peng et al., 2021; Zhan et al., 2020). Significantly, siFOXO1 treatment reduced Dex-induced expression of cleaved-caspase3, cleaved-caspase-9, and BAX, and increased the expression of Bcl2. In line with these results, flow cytometry results revealed that siFOXO1 reduced the rate of apoptotic cells, which was induced by Dex treatment, from 21.98% to 15.93%, which provided evidence that si-FOXO1 treatment attenuated Dex-induced apoptosis. These results highlight that FOXO1 may be a critical player in Dex-induced osteoblast apoptosis. However, our results were inconsistent with the results reported by other studies, which reported that GC treatment induced *β*-cell death independent of FOXO1 since siRNA-mediated downregulation of FOXO1 did not inhibit Dex-induced cell death (Kaiser et al., 2013). This difference may be attributed to the functional differences of FOXO1 in different types of cells.

FOXO1 expression may be regulated by multiple mechanisms, including its abundance, post-transcriptional modifications, nuclear-cytoplasmic shuttling, and subcellular localization (Wang et al., 2020; Peng et al., 2020). Compared to untreated cells, Dex-treatment led to decreased FOXO1 phosphorylation, increased nuclear localization, and enhanced overall expression of FOXO1. Phosphorylation is an important post-transcriptional modification of FOXO1 that regulates its subcellular localization and activity (Ju et al., 2014). The phosphorylated FOXO1 is confined to the cytosol, where it undergoes ubiquitination and proteasome-mediated degradation (Schall et al., 2015).

The PI3K/AKT signaling pathway was reported to be involved in the proliferation, differentiation, autophagy, and survival (Sawa et al., 2020; Deng et al., 2019; Jafari et al., 2019). Moreover, the activity of FOXO1 as a transcription factor is regulated *via* PI3K/AKT mediated phosphorylation (Duan et al., 2018; Goldbraikh et al., 2020; Chen et al., 2020). AKT inhibition allows FOXO1 translocation into the nucleus and activated expression of pro-apoptotic genes. We found that Dex treatment inhibited the PI3K/AKT signaling pathway by reducing the expression of p-PI3K and p-AKT. Exogenous addition of IGF-1 reversed the effects of Dex treatment by increasing the expression levels of p-PI3K, p-AKT, and p-FOXO1, while decreasing the total FOXO1 expression and its nuclear translocation. These results illustrate that Dex regulates FOXO1 expression through the PI3K/AKT signaling pathway. Specifically, Dex can induce FOXO1 entry into the nucleus by reducing the expression of p-FOXO1, thereby activating the downstream apoptotic signaling pathways.

Jing has reported that the expression of eNOS and GTPCH1 levels were up-regulated through PI3K/Akt/FOXO1, thus restoring angiogenesis in palmitic acid-impaired HUVECs (Ke et al., 2016). In vascular smooth muscle, it was reported that FOXO1 involved in the modulation the transcriptional activity of PKG (cGMP-dependent protein kinase), stimulated the production of NO, and regulated in vascular tone (An et al., 2019). These findings suggest that activation of different FOXO1-downstream targets may cause distinct cellular responses. FOXO1-mediated genes associated with NO production (such as NOS3, PRKG1, and GCH1) may be potential targets during SONFH treatment. Hence, we mined the expression levels of these genes in GSE10311 and GSE21717 (The results are presented in the File S2: Fig. 8). However, there was no significant difference in the expression of these genes before and after dexamethasone treatment. We did not find a positive regulatory relationship between PI3K/AKT/FOXO1 and NO production-related genes, which may attribute to the limited data resources related to osteonecrosis in GEO. What’s more, these researches mentioned above provide a promising direction for us to further study SONF.

*In vivo* experiments, we also found the expression of FOXO1 upregulated in SONFH rat femoral head, which was in line with the results of the cell experiment and further supported the key effect of FOXO1 in femoral head necrosis. Currently, micro-CT is the best detection system for quantitative evaluation of bone microstructure. By micro-CT scanning analysis, rats in SONFH group showed increased obvious cavity under subchondral bone, and collapse of femoral head, and Tb.Sp with the increase of apoptotic cells, but decreased Tb.N, Tb.Th, and BV/TV, when compared with the normal rats. Significantly, compared with the SONFH group, IGF-1 group showed lower empty osteocytic lacunas, and reduced apoptosis along with better trabecular shape and bone tissue integrity. These observations validated the results of the cell-based experiments.

Although this work provide evidence of PI3K/AKT/FOXO1 signaling pathway in the pathogenesis of SONFH by *in vitro* and *in vivo* experiment, the limitations of present work must be acknowledged. First, the type of the cell line *in vitro* experiment and the animal *in vivo* experiment were not enough. Second, due to few animal specimens, the protein expression levels of PI3K/AKT/FOXO1 signaling pathway in animal models were lacking. Third, experimental results still need to be verified in human femoral head specimens. We firmly believe that addressing the above limitations may provide solid evidence for the understanding, prevention and treatment of SONFH.

## Conclusion

PI3K/AKT/FOXO1 pathways are critical for Dex-induced apoptosis of osteoblasts, as observed using MC3T3-E1 cells. Our results provide evidence that in the future, FOXO1 can be used as a therapeutic target for SONFH. The exploration of mechanisms underlying the nuanced regulation of the PI3K/AKT/FOXO1 signaling pathways may help to identify effective drugs for SONFH treatment.

##  Supplemental Information

10.7717/peerj.13319/supp-1 Supplemental Information 1 The List of DEGs in SE21727

10.7717/peerj.13319/supp-2 Supplemental Information 2 Data for Figs. 2F, 3D, 5C, 6A, 6C, 6E, and 7A and the relative expression of NO production-related genes in GSE10311 and GSE21717

10.7717/peerj.13319/supp-3 Supplemental Information 3 Author Checklist - Full

10.7717/peerj.13319/supp-4 Supplemental Information 4 Western blot raw data

10.7717/peerj.13319/supp-5 Supplemental Information 5 Raw data for statistical analysis

## Additional Information and Declarations

### Data Availability

The following information was supplied regarding data availability: The raw measurements are available in the Supplementary Files and at GEO: GSE21727 and GSE10311.
