# Supplementary material for: Glucocorticoids induce osteonecrosis of the femoral head in rats via PI3K/AKT/FOXO1 signaling pathway
Source: PeerJ. 2022 May 3;10:e13319. doi: 10.7717/peerj.13319 (PMC9074886; doi:10.7717/peerj.13319)
Supplement: Supplemental Information 1 [file peerj-10-13319-s001.pdf]

GSE21727 DEGs list

GeneNam	logFC	AveExpr	t	P.Value	adj.P.Val	B
RGCC	5.83336	13.04491	60.90675	3.59E-09	6.17E-05	9.307132
FKBP5	3.293915	11.14745	53.32756	7.59E-09	6.52E-05	9.094313
C10orf10	2.890349	14.171	44.46795	2.11E-08	0.000121	8.7287
TRNP1	1.821367	11.30036	38.24183	4.94E-08	0.000198	8.35156
GPX3	4.758075	13.66704	36.81139	6.12E-08	0.000198	8.24497
NCOA3	1.073339	11.56375	36.00775	6.92E-08	0.000198	8.181124
MT2A	2.885371	14.54094	33.23574	1.09E-07	0.000267	7.936185
FIBIN	1.375857	9.958709	32.01837	1.34E-07	0.000288	7.814972
PRKAG2	1.57888	11.41892	31.06253	1.59E-07	0.000303	7.713196
MXRA5	-2.66578	13.27751	-29.1606	2.26E-07	0.000389	7.491461
MT1X	5.470112	13.18451	28.46203	2.59E-07	0.000405	7.402971
MT1A	3.417366	14.14655	27.51181	3.14E-07	0.000449	7.275909
CITED2	1.698342	13.75469	26.68613	3.72E-07	0.000492	7.158801
TSC22D3	1.154768	10.73074	25.0234	5.34E-07	0.000533	6.902152
IRS2	2.257755	10.93475	24.96779	5.40E-07	0.000533	6.893051
TEX2	1.361795	12.40374	24.3962	6.15E-07	0.000554	6.797445
ADAMTS1	2.009346	13.38948	24.01332	6.72E-07	0.000554	6.73123
CCDC69	1.136351	9.740477	23.74046	7.16E-07	0.000559	6.68294
DPYSL3	-1.09557	14.65865	-20.1885	1.77E-06	0.001127	5.958933
MT1G	1.111239	9.674931	19.27793	2.29E-06	0.001354	5.740236
ZHX3	1.396019	11.70015	18.34219	3.02E-06	0.001622	5.49878
DUSP1	1.748334	14.52699	17.57343	3.83E-06	0.001996	5.286563
SERTAD4	-1.98858	10.33017	-17.4454	3.99E-06	0.002018	5.249929
AOX1	2.637975	12.25375	16.53765	5.37E-06	0.002428	4.978875
CORO6	1.013434	9.887221	15.98507	6.48E-06	0.002651	4.803599
DRAM1	-1.33641	12.26728	-15.8521	6.79E-06	0.002651	4.760206
CRABP2	-2.53522	11.02458	-15.3118	8.23E-06	0.003072	4.578662
ADARB1	1.487528	12.08608	15.06779	8.99E-06	0.003202	4.49387
MMD	2.622263	11.44849	14.87841	9.64E-06	0.003202	4.426813
NNMT	1.057812	15.1826	14.83003	9.81E-06	0.003202	4.409505
KLF9	1.906831	13.53342	14.81352	9.87E-06	0.003202	4.403581
ZBTB16	2.010994	10.07419	14.49192	1.11E-05	0.003335	4.286462
GPR68	-1.45189	9.826413	-14.4087	1.15E-05	0.003335	4.25562
ELF1	1.058718	12.37062	14.17715	1.26E-05	0.003406	4.168552
ANGPTL4	1.705213	10.60275	14.12089	1.29E-05	0.003406	4.147125
DDIT4	2.718017	11.63112	13.8584	1.43E-05	0.003528	4.045716
EXT1	-1.15568	11.91887	-13.8265	1.44E-05	0.003528	4.033246
ALDH6A1	1.605501	11.74925	13.80241	1.46E-05	0.003528	4.023775
FMO2	2.293881	10.99293	13.65201	1.55E-05	0.003695	3.964272
SLC20A1	-1.58934	12.89558	-13.4427	1.69E-05	0.00382	3.880112
CEBPD	1.464173	14.19298	13.24154	1.83E-05	0.003983	3.797654
NFKBIA	1.235005	12.9744	13.06811	1.97E-05	0.004176	3.725336
PTX3	2.888877	13.85291	12.94819	2.07E-05	0.004339	3.674643
KLF15	1.929736	10.13133	12.86866	2.14E-05	0.004434	3.640706
MT1M	3.041528	11.09305	12.7148	2.29E-05	0.004518	3.574332
FAM107B	-1.00203	10.80045	-12.6808	2.32E-05	0.004533	3.559547
SAMHD1	1.176696	9.787015	12.51481	2.50E-05	0.004817	3.486602
PRRX2	-1.21328	12.36434	-12.1828	2.89E-05	0.005197	3.337225
ACSL1	1.128096	10.6274	12.17224	2.90E-05	0.005197	3.332376
MMP7	4.876098	11.53109	12.12262	2.97E-05	0.005227	3.309613
FOS	1.461788	10.62138	12.0482	3.07E-05	0.00533	3.275267
FMO3	1.724511	10.97843	11.95193	3.21E-05	0.005405	3.230464
GALNT15	3.517637	11.44587	11.75735	3.51E-05	0.005683	3.138618
EGR2	-1.22305	9.892491	-11.7387	3.54E-05	0.005683	3.129727
SSH2	1.301978	10.54867	11.65448	3.68E-05	0.005856	3.089348

CD248	-1.67412	12.84892	-11.5786	3.81E-05	0.005958	3.052682
AK1	-1.47159	12.3794	-11.5334	3.90E-05	0.006031	3.03073
ELL2	1.280405	11.70126	11.49135	3.97E-05	0.006054	3.010179
GLIPR1	-1.06204	12.86256	-11.2459	4.47E-05	0.006347	2.88863
ENG	-1.64156	11.3163	-11.2212	4.52E-05	0.00637	2.87622
CFLAR	1.135292	11.41243	11.13338	4.72E-05	0.006504	2.831869
MAOA	2.462077	11.9866	11.11538	4.76E-05	0.006504	2.822732
SERPINA3	4.658756	12.92522	11.09788	4.80E-05	0.006504	2.813835
KCNN4	-1.56658	10.40815	-10.8548	5.42E-05	0.006935	2.688592
CGNL1	-1.23614	10.49416	-10.8238	5.50E-05	0.006935	2.672376
CAT	1.141995	12.23529	10.80885	5.54E-05	0.006935	2.664543
SQRDL	1.191265	11.23569	10.79173	5.59E-05	0.006935	2.655565
PHLDA1	-1.94405	11.65683	-10.7215	5.79E-05	0.006958	2.618526
GREM1	-3.26597	12.20858	-10.6712	5.94E-05	0.007088	2.591881
WASF3	1.55293	11.96676	10.63824	6.04E-05	0.007158	2.574323
SPSB3	1.031887	12.24716	10.54479	6.34E-05	0.007221	2.524227
IER3	-1.93103	11.82779	-10.5416	6.35E-05	0.007221	2.522484
ANGPTL7	4.81869	12.65793	10.44958	6.65E-05	0.007353	2.472683
PDK4	2.851057	10.84469	10.41774	6.76E-05	0.007353	2.455332
RHOBTB3	1.520273	13.30879	10.38944	6.86E-05	0.007353	2.439862
IMPA2	3.020632	12.27583	10.21079	7.54E-05	0.00766	2.341128
MLLT11	-1.88337	11.73321	-10.1667	7.72E-05	0.007674	2.316449
ADAM19	-1.21251	11.11012	-10.1555	7.76E-05	0.007674	2.310192
WBP1L	1.439082	11.88257	9.924299	8.79E-05	0.008116	2.178842
FOXO1	2.132476	11.40987	9.840299	9.20E-05	0.008302	2.13031
DCXR	1.125581	10.72186	9.794199	9.43E-05	0.008353	2.103488
VCAM1	-1.72218	10.50796	-9.75057	9.66E-05	0.008425	2.077977
VCAN	-1.11143	13.35419	-9.52254	0.00011	0.009016	1.942675
SRPX	1.862033	14.06047	9.320524	0.000123	0.009469	1.819953
PLA2G4A	-1.23795	10.34417	-9.30018	0.000124	0.009469	1.807443
ABCA6	1.451883	9.948854	9.291719	0.000125	0.009469	1.80223
DHRS3	1.566267	11.73917	9.251926	0.000128	0.009469	1.777654
CNTNAP1	-1.02012	11.12651	-9.24544	0.000128	0.009469	1.773636
LOXL1	-1.06575	10.8852	-9.24017	0.000129	0.009469	1.770372
MAP3K7C	-2.00775	11.94175	-9.21014	0.000131	0.009469	1.751728
TGFB3	-1.39563	10.49168	-9.20433	0.000132	0.009469	1.748112
EXOSC10	1.026568	11.55721	9.19703	0.000132	0.009469	1.74357
STC2	3.391577	13.66107	9.191133	0.000133	0.009469	1.739896
FBXO32	1.050545	11.39058	9.189628	0.000133	0.009469	1.738958
NFE2L2	1.182926	13.09266	9.071128	0.000142	0.009817	1.664607
FAM46B	1.464587	10.39988	9.015323	0.000147	0.00991	1.629249
EBF1	1.011103	12.0165	8.854678	0.000162	0.010186	1.526215
CXXC5	-1.1032	12.31579	-8.8354	0.000164	0.010186	1.513721
SLC14A1	-2.08432	10.97883	-8.83473	0.000164	0.010186	1.513288
EVA1C	1.413713	10.65012	8.815477	0.000165	0.010186	1.500785
AGTR1	1.092094	10.27497	8.718853	0.000175	0.010615	1.43762
ANOS1	-1.11414	9.728342	-8.70062	0.000177	0.010621	1.425619
LEPR	1.241178	10.49624	8.650054	0.000183	0.010804	1.392217
GADD45B	1.900561	11.96916	8.594108	0.000189	0.011005	1.355031
APOD	3.088488	10.90506	8.592385	0.00019	0.011005	1.353881
SLC26A6	2.292542	11.85053	8.563338	0.000193	0.01102	1.334476
ZCCHC6	1.380265	12.20255	8.562976	0.000193	0.01102	1.334233
MTHFD2	1.788459	11.45735	8.521052	0.000198	0.011083	1.306109
CMTM6	1.058154	12.06917	8.498717	0.000201	0.011125	1.291069
CNKSR3	1.056095	11.0535	8.445875	0.000208	0.011209	1.255333
DKK1	2.878506	11.81317	8.442261	0.000208	0.011209	1.25288
RCAN1	-1.01542	10.86096	-8.31556	0.000225	0.011629	1.166259
CH25H	-1.48185	10.03623	-8.2717	0.000232	0.011728	1.135969

LMNA	-1.29864	12.89059	-8.21668	0.00024	0.01199	1.097756
MAP1LC3(	2.830923	10.78606	8.146796	0.000251	0.012291	1.048858
LRRC17	-1.16172	9.957483	-8.13794	0.000253	0.012291	1.04263
HECW2	-1.28061	11.0129	-8.09872	0.000259	0.012384	1.014984
FRZB	1.211281	10.04162	8.085741	0.000261	0.012384	1.005808
MAP3K5	1.025175	10.42103	8.060498	0.000266	0.012465	0.987917
FZD4	1.340157	11.44976	8.039902	0.000269	0.012541	0.97328
GFPT1	-1.07368	11.51109	-8.01872	0.000273	0.012541	0.958186
GLRX	1.287283	13.67813	7.820309	0.000311	0.013379	0.814964
BHLHE40	-1.85146	11.28214	-7.76259	0.000323	0.013686	0.772652
FOXQ1	-1.32639	10.03771	-7.70425	0.000336	0.013894	0.72959
NT5DC2	-1.17206	11.39587	-7.69505	0.000339	0.013947	0.722768
ADH1A	2.888673	10.9113	7.653	0.000348	0.014082	0.691502
HSPA5	-1.36994	11.20211	-7.62668	0.000355	0.014223	0.671849
MYLK	-1.2639	12.24334	-7.61775	0.000357	0.014223	0.665168
PAPLN	1.01466	10.30086	7.585257	0.000365	0.014359	0.640794
NEXN	1.496137	12.01207	7.489304	0.00039	0.015014	0.56825
KRT19	-1.71234	10.66852	-7.45065	0.0004	0.015219	0.538781
TMEM119	-1.75351	12.96827	-7.44865	0.000401	0.015219	0.537254
PSME4	-1.13414	11.15966	-7.42117	0.000409	0.015245	0.516214
FPR1	1.291485	9.925165	7.311022	0.000442	0.015697	0.431166
COL7A1	2.215371	11.06803	7.225817	0.000469	0.016027	0.364574
GPR88	-1.07717	9.670291	-7.11767	0.000507	0.016565	0.279031
MRAP2	-1.05826	10.47409	-7.08112	0.000521	0.016639	0.249849
ARHGEF1(	1.168663	10.62822	7.080761	0.000521	0.016639	0.249565
THBS2	-1.29526	15.04001	-7.07964	0.000521	0.016639	0.24867
CBS	1.504565	11.54058	7.025545	0.000542	0.016849	0.205233
COL8A2	-1.12621	11.7916	-7.01567	0.000546	0.01691	0.197276
TIMP4	1.493552	10.35145	6.997299	0.000554	0.01701	0.182436
IGF2BP2	1.057578	12.25617	6.99043	0.000556	0.017011	0.17688
FKBP14	-1.26815	11.52595	-6.9524	0.000572	0.01724	0.146031
STOM	1.581083	12.9367	6.836995	0.000623	0.018077	0.051499
IL6	-1.90406	10.3496	-6.82279	0.00063	0.018168	0.039767
KDELR3	-1.45532	11.80447	-6.79905	0.000641	0.018307	0.020111
SERPINH1	-1.51078	12.79615	-6.75865	0.000661	0.018747	-0.01347
CAMK2N1	1.037433	12.11056	6.726251	0.000678	0.019024	-0.04053
RDH10	-1.59544	11.56227	-6.63482	0.000727	0.019596	-0.11749
DDIT4L	-1.93538	10.20463	-6.5816	0.000757	0.02021	-0.16272
ADRA1B	1.538872	9.930498	6.576491	0.00076	0.02021	-0.16708
DHCR24	1.224191	11.43719	6.551264	0.000775	0.020454	-0.18863
GPM6B	2.6042	10.94916	6.538714	0.000782	0.020523	-0.19938
GCHFR	1.913165	11.1061	6.523465	0.000792	0.020704	-0.21247
APCDD1L	-1.28442	13.17802	-6.42237	0.000857	0.021669	-0.29989
MIOS	1.584223	10.99126	6.421173	0.000858	0.021669	-0.30093
CD302	1.26066	11.47421	6.414735	0.000862	0.021747	-0.30654
MYC	1.221185	11.43148	6.375076	0.000889	0.022145	-0.34118
CADM1	-1.05926	9.780624	-6.29541	0.000948	0.022669	-0.41131
SSB	1.23595	13.02326	6.28791	0.000953	0.022669	-0.41795
CDKN1C	1.163015	10.08291	6.276962	0.000962	0.022743	-0.42766
EGR1	-1.47988	11.5073	-6.22907	0.000999	0.023264	-0.47027
ITGA10	1.450187	11.78275	6.225846	0.001002	0.023293	-0.47315
P4HA2	-1.16995	12.39094	-6.18731	0.001034	0.023804	-0.50765
FABP4	1.428732	10.37965	6.167759	0.00105	0.023996	-0.52522
QPCT	1.273367	12.15675	6.107948	0.001103	0.024729	-0.57926
SETMAR	1.004662	11.14668	6.073022	0.001135	0.025185	-0.61101
LFNG	-1.23142	9.866969	-6.06779	0.001139	0.025261	-0.61579
RGS4	-2.50404	11.60643	-5.96088	0.001245	0.026836	-0.71396
SAT1	1.276374	14.57925	5.863569	0.001351	0.028096	-0.80454

SESN1	1.021803	11.11837	5.860714	0.001354	0.028096	-0.80722
SCD	-2.52574	11.55318	-5.8222	0.001399	0.028805	-0.84341
GPC4	-1.10148	10.95054	-5.81045	0.001413	0.028956	-0.85449
METTL7A	1.053573	10.45387	5.799323	0.001426	0.029078	-0.86499
FAXDC2	1.143533	10.59364	5.743963	0.001495	0.02984	-0.9175
ACSS1	1.18705	9.870823	5.707986	0.001542	0.030423	-0.95184
CAV1	1.083171	11.96611	5.651712	0.001619	0.031274	-1.00587
BCL6	1.378591	13.25517	5.61495	0.001672	0.031787	-1.04139
PTGER2	1.194482	10.48103	5.503369	0.001844	0.033631	-1.15029
MMP1	-1.14897	9.618347	-5.47832	0.001885	0.033989	-1.17496
LDB2	-2.09211	11.82619	-5.44518	0.001942	0.034346	-1.20773
FST	-1.30904	12.187	-5.43449	0.00196	0.034434	-1.21834
NFIL3	1.459173	10.98071	5.365661	0.002085	0.035504	-1.28697
CKAP4	-1.21277	12.81424	-5.36526	0.002086	0.035504	-1.28737
TNFRSF12	-1.58985	11.52327	-5.33871	0.002137	0.035998	-1.31402
THY1	-1.24094	14.37154	-5.33012	0.002153	0.036102	-1.32266
ADAMTSL	-1.53103	10.37644	-5.24764	0.002321	0.037673	-1.40614
EVA1A	-1.09335	10.67433	-5.24719	0.002322	0.037673	-1.40661
CCL2	-1.77448	12.69961	-5.23235	0.002354	0.037938	-1.42173
PLAT	-1.84577	11.81647	-5.19258	0.002442	0.038609	-1.46241
ITGA11	-1.09633	13.74921	-5.1752	0.002481	0.038794	-1.48025
CCL8	-1.12028	9.738126	-5.17495	0.002482	0.038794	-1.48051
NDRG1	1.266681	12.83982	5.141682	0.002559	0.039412	-1.51478
CAP2	-1.16478	11.57269	-5.09558	0.002672	0.040406	-1.56254
MYH11	-1.02981	10.21557	-5.08678	0.002694	0.040704	-1.57169
TUBB3	-1.48888	11.61059	-5.02942	0.002843	0.041746	-1.63161
CPA4	-1.07139	9.595874	-5.02761	0.002848	0.041746	-1.6335
CMTM8	1.246628	10.69122	5.021679	0.002864	0.041898	-1.63972
CDO1	1.726673	11.1978	4.943149	0.003085	0.043845	-1.72259
BDKRB1	-1.41812	9.822917	-4.92439	0.003141	0.044163	-1.74252
MARCKSL	-1.04277	11.00104	-4.87968	0.003279	0.045357	-1.79021
KDELR2	-1.03048	12.67706	-4.84212	0.0034	0.046287	-1.83051
DUSP23	1.303572	11.93262	4.822254	0.003466	0.046666	-1.8519
TXNIP	2.08121	14.44095	4.802852	0.003532	0.04704	-1.87285
RGMA	1.147993	11.2297	4.774303	0.003632	0.04749	-1.90378
LAMB1	1.075027	12.90218	4.692357	0.003936	0.04929	-1.99322
NTM	-1.32961	10.67996	-4.68636	0.003959	0.049474	-1.9998
TSPAN13	-1.22629	10.35009	-4.67447	0.004006	0.049879	-2.01287
