# Supplementary material for: Glucocorticoids induce osteonecrosis of the femoral head in rats via PI3K/AKT/FOXO1 signaling pathway
Source: PeerJ. 2022 May 3;10:e13319. doi: 10.7717/peerj.13319 (PMC9074886; doi:10.7717/peerj.13319)
Supplement: Supplemental Information 2 [file peerj-10-13319-s002.pdf]

Figure 1: Flow cytometric analysis of apoptosis rates of MC3T3-E1 cells upon Dex treatment.

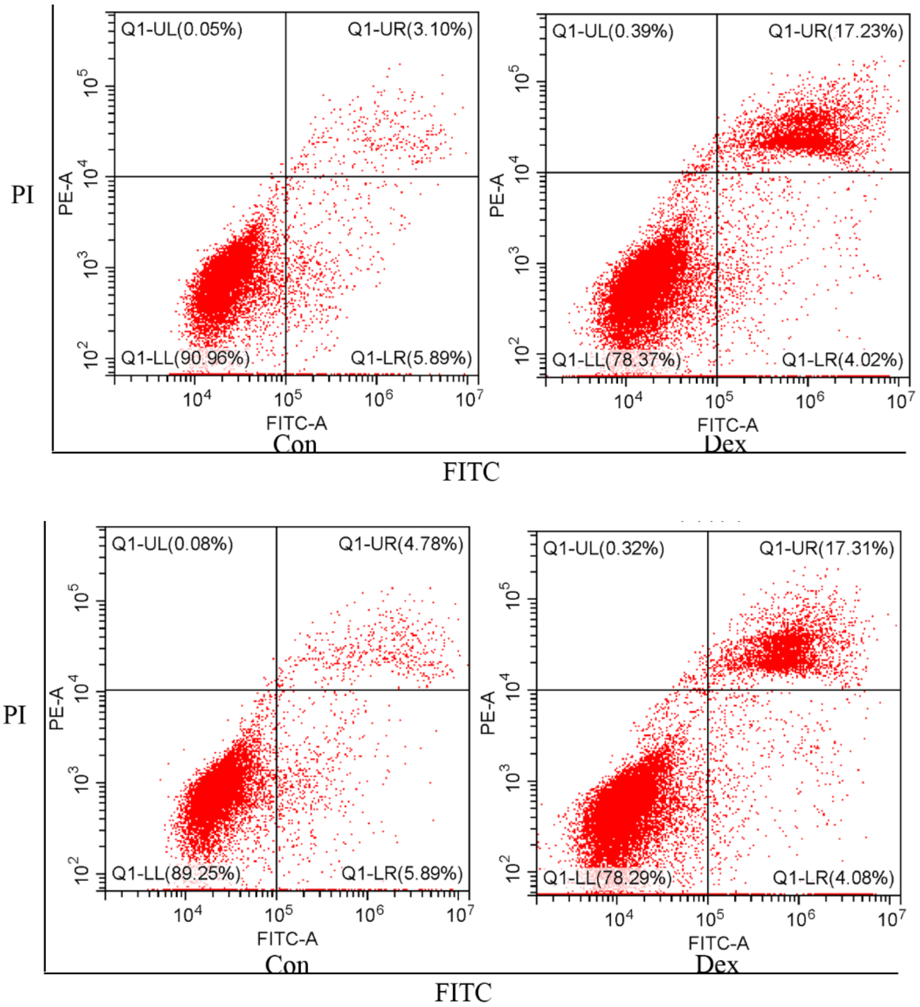

Figure 2: MC3T3-E1 cell apoptosis after 48 h Dex and Foxo1 siRNA treatment as determined using FITC-PI/Annexin V staining.

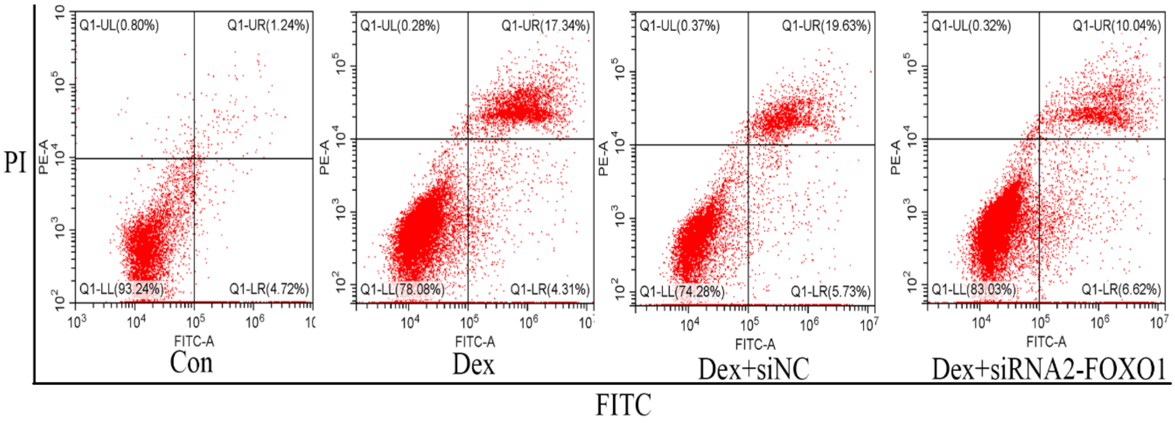

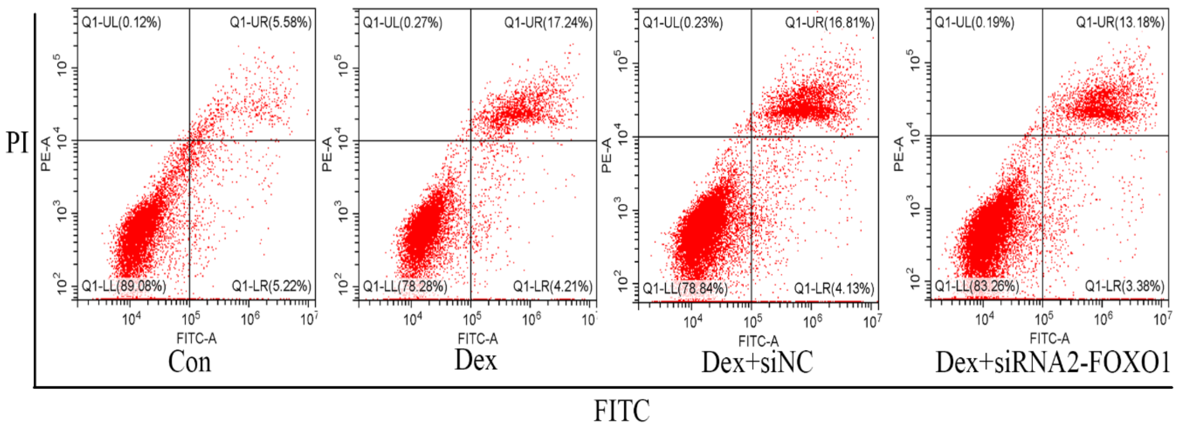

Figure 3: The distribution and expression of FOXO1 protein in the MC3T3-E1 treated with Dex was evaluated by immunofluorescence.

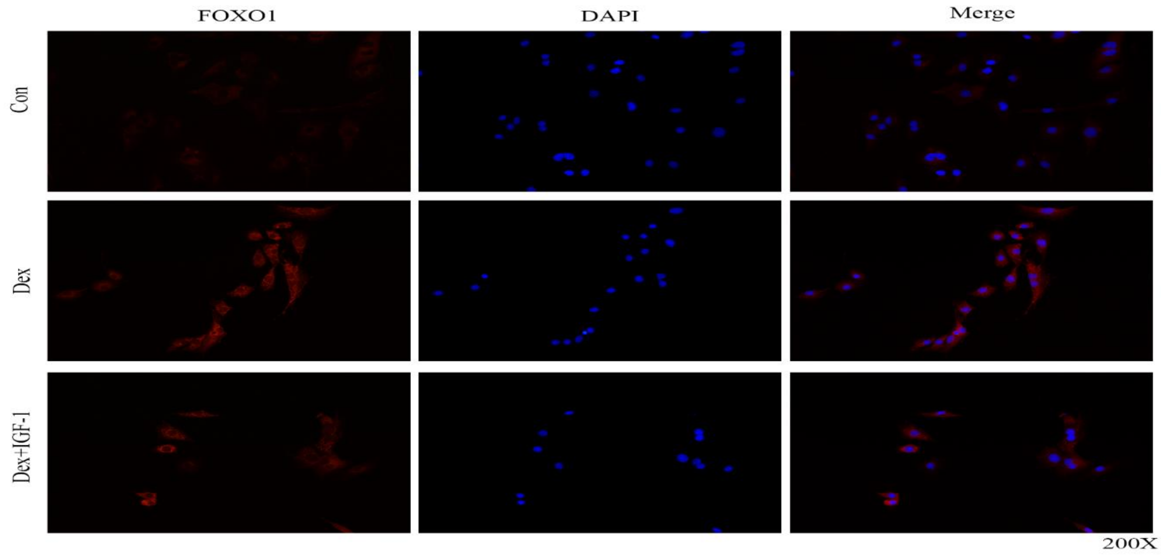

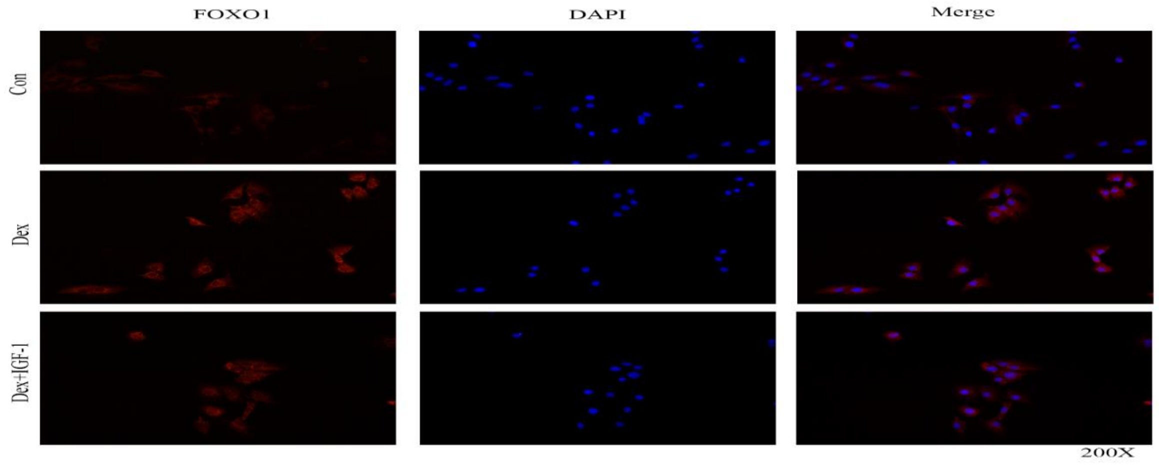

Figure 4: HE staining of femoral heads of rats in the Normal, MPS, and IGF-1+ MPS

group; green arrows indicate empty osteocytic lacunas.

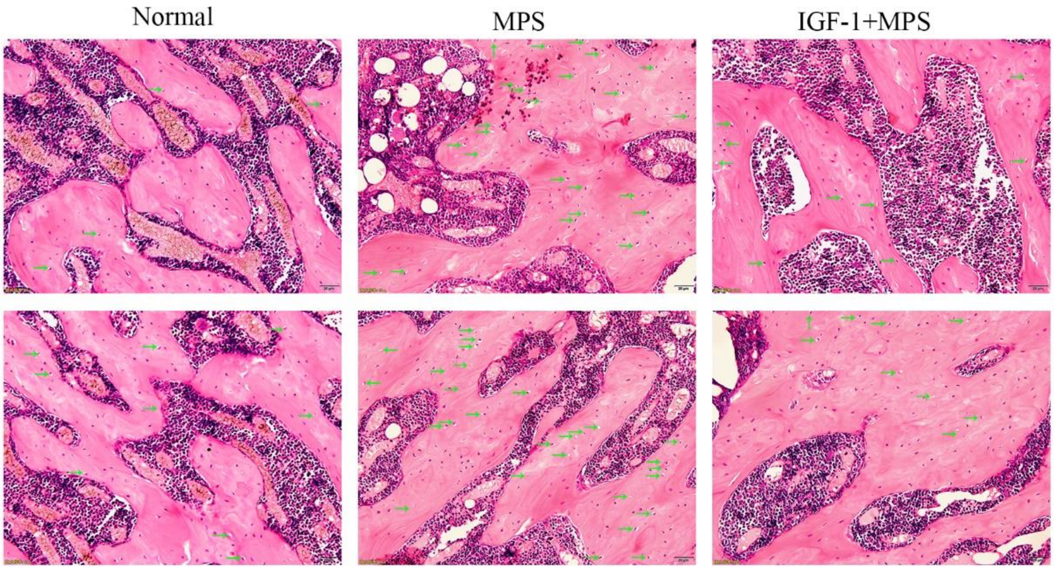

Figure 5: Tunel staining to detect apoptosis cells; green arrows indicate positive apoptosis cells.

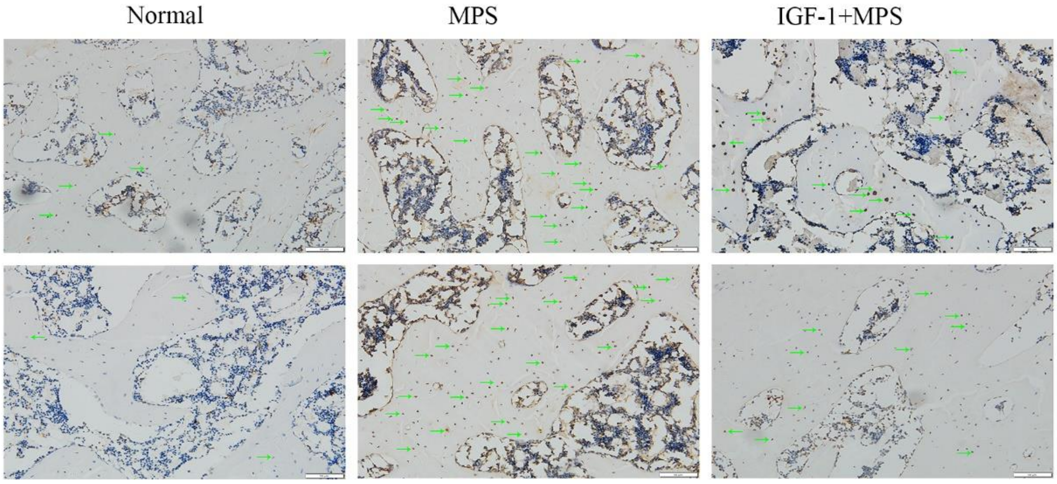

Figure 6: Immunohistochemistry to evaluate the expression of FOXO1 in femoral heads of rats; green arrows indicate FOXO1-positive cells that are stained as brown dots.

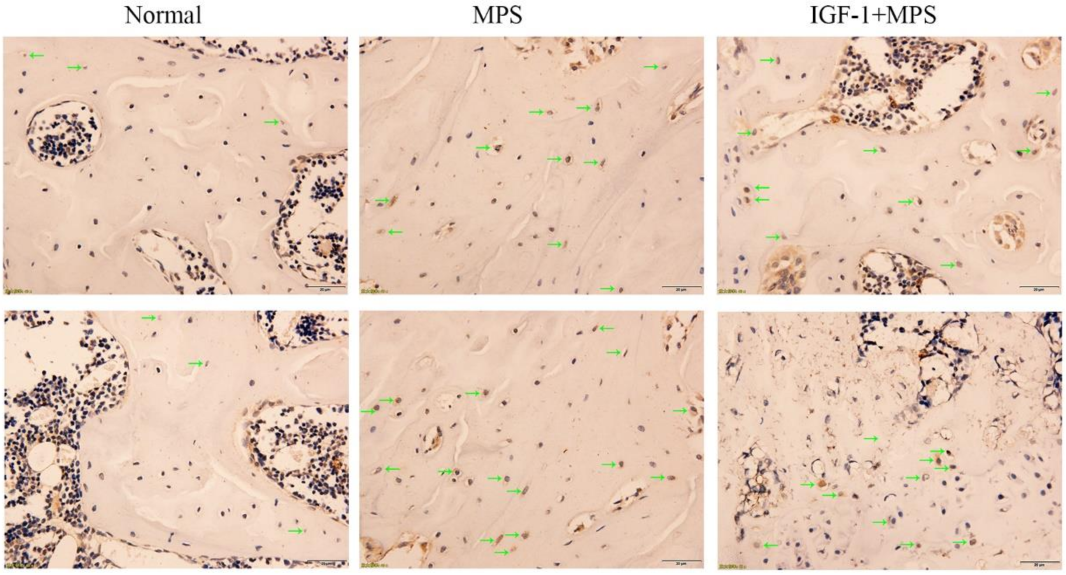

Figure 7: Micro-CT analysis of femoral heads of rats in the Normal, MPS, and IGF-1+ MPS group.

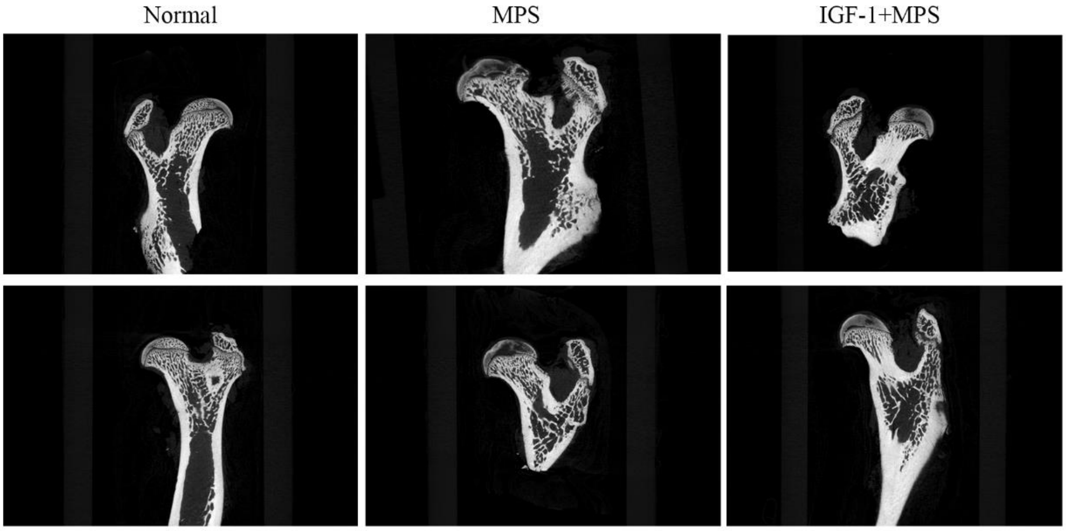

Figure 8: The mRNA expression of NOS3, GCH1,and PRKG1 in GSE10311 and GSE21727.

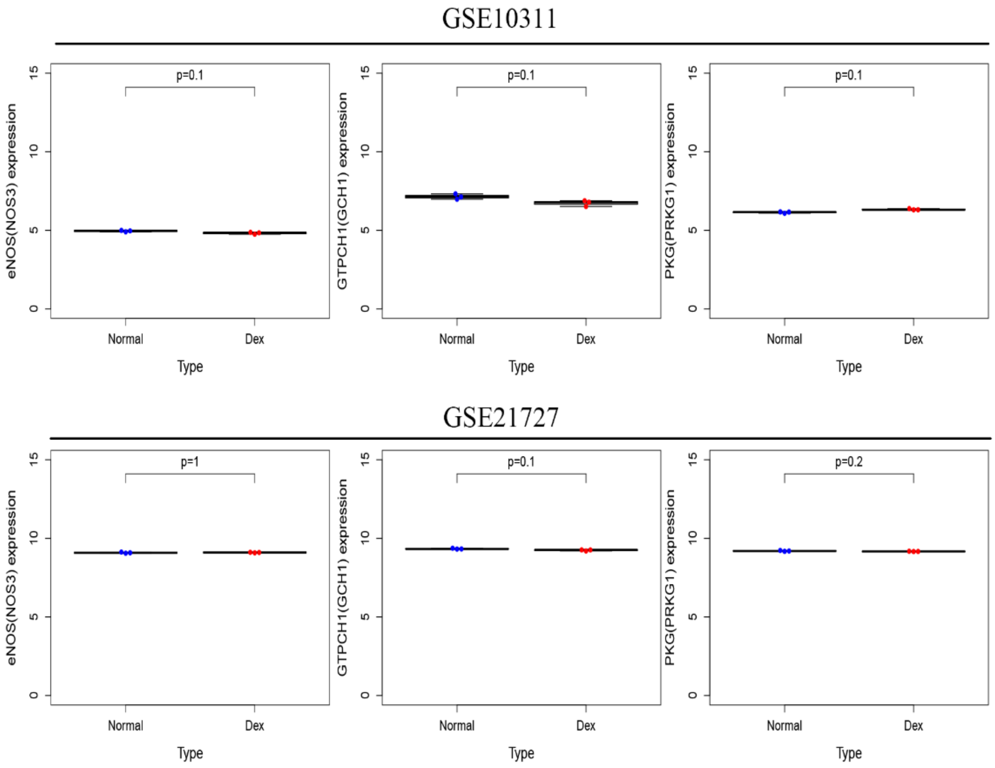
GSE10311
eNOS(NOS3) expression
GTPCH1(GCH1) expression
PKG(PRKG1) expression
p=0.1
p=0.1
p=0.1
Normal
Dex
Type
GSE21727
p=1
p=0.1
p=0.2
0
5
10
15
