# Supplementary figures and images for: Glucocorticoids induce osteonecrosis of the femoral head in rats via PI3K/AKT/FOXO1 signaling pathway
Source: PeerJ. 2022 May 3;10:e13319. doi: 10.7717/peerj.13319 (PMC9074886; doi:10.7717/peerj.13319)

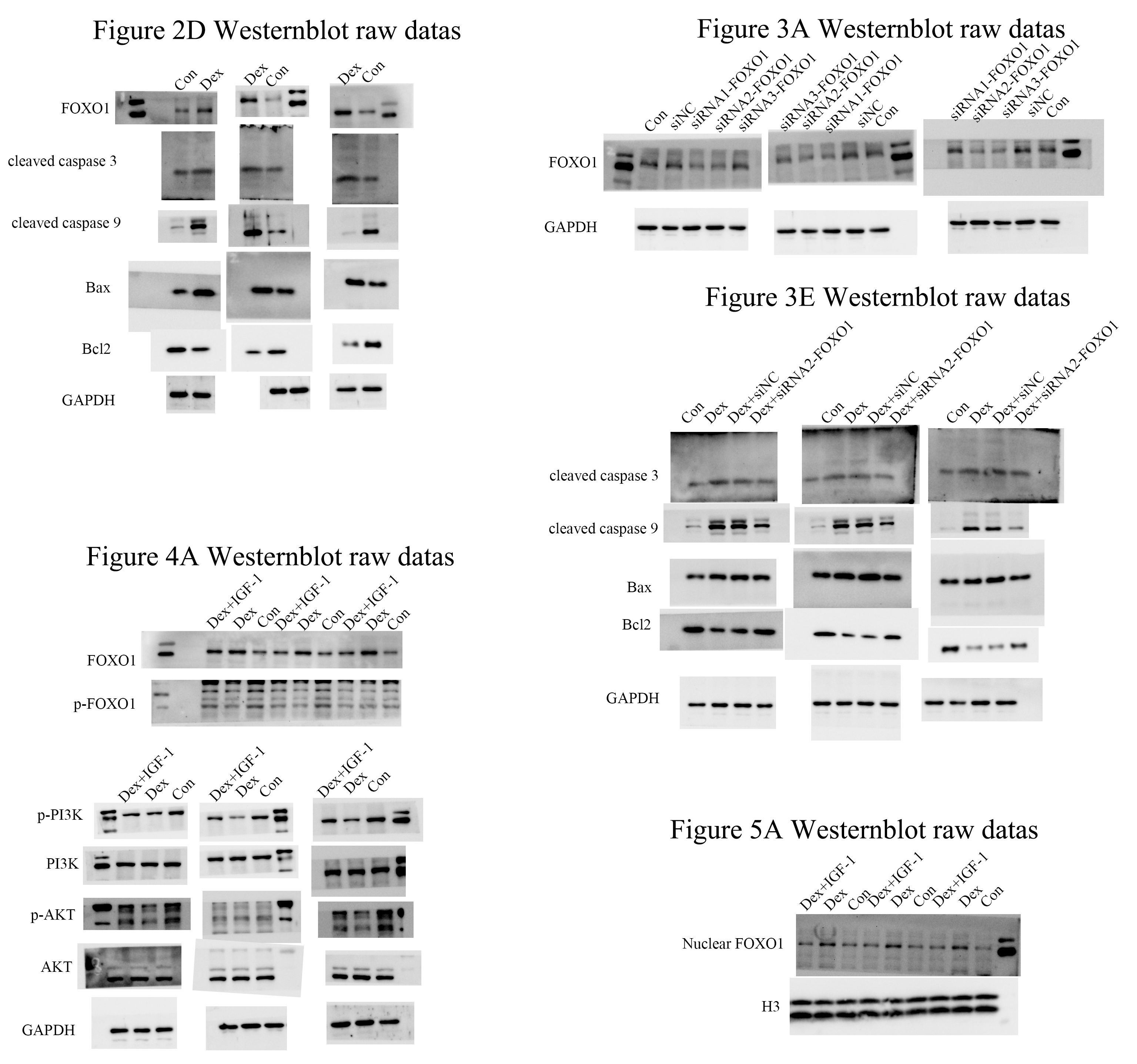

Supplement: Supplemental Information 4 [file peerj-10-13319-s004.png]
